# Is unemployment in young adulthood related to self-rated health later in life? Results from the Northern Swedish cohort

**DOI:** 10.1186/s12889-017-4460-z

**Published:** 2017-05-30

**Authors:** Fredrik Norström, Urban Janlert, Anne Hammarström

**Affiliations:** 1 Department of Public Health and Clinical Medicine, Epidemiology and Global Health, Umeå University, SE 901 87 Umeå, Sweden; 2 Department of Public Health and Caring Sciences, Uppsala University, Uppsala, Sweden

## Abstract

**Background:**

Many studies have reported that unemployment has a negative effect on health. However, little is known about the long-term effect for those who become unemployed when they are young adults. Our aim was to examine how unemployment is related to long-term self-rated health among 30 year olds, with an emphasis on how health differs in relation to education level, marital status, previous health, occupation, and gender.

**Methods:**

In the Northern Swedish Cohort, 1083 teenagers (~16 years old) were originally invited in 1981. Of these, 1001 participated in the follow-up surveys in 1995 and 2007. In our study, we included participants with either self-reported unemployment or activity in the labor force during the previous three years in the 1995 follow-up so long as they had no self-reported unemployment between the follow-up surveys. Labor market status was studied in relation to self-reported health in the 2007 follow-up. Information from the 1995 follow-up for education level, marital status, self-reported health, and occupation were part of the statistical analyses. Analyses were stratified for these variables and for gender. Analyses were performed with logistic regression, G-computation, and a method based on propensity scores.

**Results:**

Poor self-rated health in 2007 was reported among 43 of the 98 (44%) unemployed and 159 (30%) of the 522 employed subjects. Unemployment had a long-term negative effect on health (odds ratio with logistic regression 1.74 and absolute difference estimates of 0.11 (G-computation) and 0.10 (propensity score method)). At the group level, the most pronounced effects on health were seen in those with upper secondary school as their highest education level, those who were single, low-level white-collar workers, and women.

**Conclusions:**

Even among those becoming unemployed during young adulthood, unemployment is related to a negative long-term health effect. However, the effect varies among different groups of individuals. Increased emphasis on understanding the groups for whom unemployment is most strongly related to ill health is important for future research so that efforts can be put towards those with the biggest need. Still, our results can be used as the basis for deciding which groups should be prioritized for labor-market interventions.

**Electronic supplementary material:**

The online version of this article (doi:10.1186/s12889-017-4460-z) contains supplementary material, which is available to authorized users.

## Background

It is generally agreed that unemployment is related to poor health [1–3]. It has been debated whether unemployment causes poorer health or if poorer health among unemployed individuals can be solely explained by poor health increasing the risk of getting unemployed. The most common view is that unemployment in fact causes poorer health [3], but there are also a few studies arguing against this [4]. The study context has been shown to have a major role in explaining how unemployment is related to poorer health [2], so it is possible, therefore, that both those who argue for and against the causality link might be partly correct.

Less is known about the long-term effects of unemployment on health. The few studies that have examined this are well in agreement that unemployment is related to poorer long-term health [5–9] as well as other social adversities such as lower income [10]. A long-term negative health effect from youth unemployment has been shown in studies using the Northern Swedish Cohort [11] in the form of psychological symptoms [6, 8, 9], somatic symptoms [5, 6], and high blood pressure [7] in follow-ups of 16 year olds at 30 and 42 years of age. It is rare for studies to look at long-term follow-ups of health effects from unemployment at a later age. Strandh et al., using data from the Northern Swedish cohort, could not confirm a long-term effect on psychological symptoms from unemployment at the age of 30 years in a 12-year follow-up [8].

Many variables have been included as part of the statistical analysis for studies of the relationship between unemployment and health, the most common being gender, age, education level, marital status, household income, geographic location, and social network/support [2]. In studies of the relationship between unemployment and health, most of these variables are commonly only of interest as potentially confounding variables and are not presented with stratified estimates for each outcome of the factors. Gender, age, and geographic location were actually the only factors where results were reported for each outcome in at least 5 of the 41 studies in a recent review [2].

In previous studies with results presented on the group level, the effect on health from becoming unemployed has usually differed between groups [2]. The groups most disfavored by unemployment often vary depending on when the study was performed and the target population that was studied. Only for manual workers (compared with non-manual workers) [12–14], those unemployed due to health reasons (compared with those unemployed for other reasons) [15], and those with a weak social network (compared with those with a strong social network) [16, 17] has a greater risk for poor health been unequivocally demonstrated. However, these conclusions are based on only a few studies, so it can still be questioned whether similar conclusions can be drawn for any context or population.

There is a need for gendered and contextualized analyses as studies in the field have shown that unemployment could have various impacts on the health status of men and women [12, 18, 19]. Here, Raewyn Connells theories about how the patterned relations between men and women that form gender as a social structure, could be useful [20]. According to her theory, gender relations are on a structural level integrated into the labor market and in this way, different labor market conditions are constructed for men and women. Also for other groups, such as age and education, stratified results have been inconclusive, and they seem to depend on the study context. For some groups, there have even been results indicating no health effect or even potentially positive health effects from unemployment, e.g. for Spanish women [14] and for Swedes with only a primary-school education [21]. Thus it is not usually possible to draw general conclusions about how unemployment affects different groups [2].

Potentially confounding variables must be considered in the analysis of the effect on health from unemployment, and they need to be handled well in the statistical model. One of the keys to having good estimates of the health effect from unemployment is being able to overcome the problem arising from health selection, which appears due to people who become unemployed being more likely to have previous health problems than those who remain employed. For long-term effects due to unemployment, caution about unemployment during the follow-up period is needed to avoid interpretations related to a more recent unemployment experience. In previous studies this has been handled in the statistical analysis model [5–9], while a novel approach used in our study is to only include those without unemployment in the follow-up period. The health effect from being re-employed has been studied many times [1], but such research has had a different focus than our study, which is on the lasting health effect later in life due to unemployment.

Thus, our aim was to examine how unemployment is related to long-term self-rated health among 30 year olds, with an emphasis on how health differs in relation to education level, marital status, previous health, occupation, and gender.

## Methods

### Study design and participants

The Northern Swedish Cohort was initiated in 1981. In that study, all pupils, most of whom were born in 1965, who were in their last year of compulsory school in a middle-sized town in Northern Sweden were invited to participate. For the cohort, there have been four follow-ups (1983, 1986, 1995, and 2007) [11]. Comprehensive questionnaires were distributed at the initial time of inclusion and at the four follow-ups, and the response rates have been very high, ranging from 1080 (99.7%) of 1083 invited individuals in the baseline investigation to 1010 individuals at the latest follow-up in 2007 (94.3% of those still alive). Further information about the Northern Swedish Cohort is available elsewhere [11].

### Inclusion criteria

**Fig. 1:**
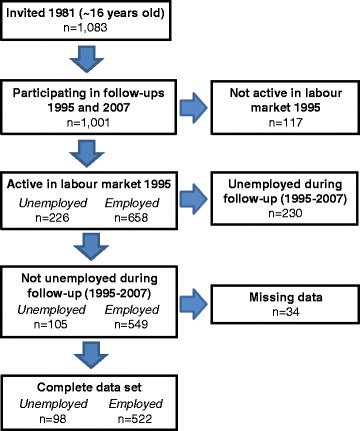
Flow chart of study participants. School leavers (~16 years of age) in a middle-sized town in Northern Sweden were invited in 1981. Follow-up surveys were conducted in 1995 and 2007. Participants were defined as active in the labor market 1995 if they were *unemployed* or *employed*. Requirements for being defined as unemployed and employed 1995 and not unemployed during follow-up are given in detail in the methods section

In our study, survey data from the follow-ups in 1995 (when the participants were ~30 years of age) and 2007 (when the participants were ~42 years old) were used, which were available for 1001 individuals. We restricted our selection of individuals to the 654 participants who were active in the labor market at the follow-up in 1995 and who had no self-reported unemployment in between the follow-ups of 1995 and 2007. We used the inclusion criteria to detect differences in health on a longer time perspective due to unemployment and not due to unemployment spells between the follow-ups of 1995 and 2007. For all of our analyses, we required eligible responses on all of our candidate variables, leading to the exclusion of 34 individuals and resulting in a final selection of 620 individuals. Our final selection of individuals corresponded to 58% of those invited and still alive from the original cohort at the time of the 2007 follow-up. The definitions of “active in the labor market” and “no self-reported unemployment in between follow-ups” are given in the section “Definition of labor market status”. A flow chart of the inclusion criteria is shown in Fig. 1.

### Questionnaire data

From the 2007 questionnaire, besides using no self-reported unemployment as an inclusion criterion, only our outcome variable, which measures self-rated health through three response alternatives (“good”, “fairly good”, and “poor”) was used. For self-rated health, “fairly good” and “poor” were used to represent poor health, and “good” was used to represent good health. From the 1995 questionnaire, the same question was used and recoded identically, but questions about labor market status, education level, marital status, occupation, social support, cash margin, smoking, alcohol intake, weight, and height were also used. The labor market status variables were chosen to define the exposure to unemployment, while other variables were chosen due to them being listed among the most commonly used variables in studies similar to ours in a recent review [2], which hints that they are potential confounders in the relationship between health and labor market status, and because these variables were collected in the Northern Swedish Cohort [11]. Self-rated health in 1995 allowed the health difference due to current/recent unemployment in 1995 to be taken into account in our analyses.

### Definition of labor market status

For labor market status, unemployment was used as the exposure and compared with employment as the non-exposure. We defined the participants’ labor market status based on self-reported labor market status during the last three years using questionnaire responses from 1995. In the questionnaire, a tick for employment status(es) was given for each of the half years between autumn 1986 and autumn 1995 (the time period since the previous follow-up). From the listed employment statuses in the questionnaire, the alternatives “Full-time employment”, “Part-time (20–39 hours a week) employment” and “Labor market measure” were defined as “Employed”, the alternative “Unemployed” was defined as “Unemployed”, and the alternatives “University/high-school”, “Other education”, “Casual job (< 20 hours a week)”, “Sick leave”, “On parental leave”, and “Other” were defined as not being active in the labor market. A tick as unemployed during any of the six half-year periods between autumn 1992 and autumn 1995 defined the participant as unemployed in our study. Participants not defined as unemployed were defined as employed in our study if they had a tick for any of the “employed” alternatives for at least three half-year periods during the same time period of 1992 to 1995.

To be considered to have no unemployment between follow-ups, which was a criterion for being included in our study, participants’ responses to employment statuses between spring 1996 and autumn 2007 in the 2007 questionnaire were used. Participants were defined as having no unemployment during this time period if besides no unemployment being reported they also had at least three ticks for alternatives defined as employed during the period between spring 1996 and autumn 2007. Thus, we compared those with unemployment (the exposed group) during the ages of 28–30 years old with those who were employed (the reference group) during these ages, and we only made comparisons between individuals with no unemployment between 30 and 42 years of age. Requiring employment during at least 12 of the 24 time periods would have decreased our sample from 620 to 608 individuals, thus having only a small influence on the results.

### Potential confounding variables at age 30

Education level was divided into three groups – at most 2 years of secondary education, 3–4 years of secondary education (referred to as “upper secondary education”), and university studies (bachelors degree or completion of other education at higher level) – with at most 2 years of secondary education being the reference group. For marital status, the alternatives “living with wife/husband” and “living with cohabitant/partner” were defined as “married” and were used as the reference group, and the other alternatives of “alone”, “with a friend”, and “other” were defined as “single” and used as the exposure group. A socio-economic index was derived for each respondent from their description of their occupation based on the nomenclature used by Statistics Sweden [22], with blue-collar workers (codes 11–22 and 89) as the reference group and low-level white-collar workers (codes 31–36) and medium- to high-level white-collar workers (codes 42–79) as the exposure groups. For gender, men were used as the exposure group.

For measuring social support, we used the Availability of Social Integration (AVSI) and Availability of Attachment (AVAT) instruments, which are part of the Interview Schedule for Social Interaction [23]. The AVSI consists of four questions with six response alternatives, and the AVAT consists of six questions with four alternatives. In both cases the questions are summed together for a maximum score of 24. For the AVSI, we used a cut-off of 13 or lower as the reference group, and for the AVAT we used a cut-off of 10 or higher as the reference group. For cash margin, the availability of 13,000 SEK (corresponding to 1366 euro on 28 March 2017) within a week was used as the reference group, and no availability of 13,000 SEK within a week was used as the exposure. For smoking, “not a current smoker” was used as the reference group and was compared with i) “smoking at most 10 cigarettes a day” or “smoking pipe or smoking cigar”, and ii) “smoking more than 10 cigarettes a day”.

The total alcohol consumption in centiliters of pure alcohol per year for a study participant was calculated based on six questions, one for frequency and one for the amount of intake on each drinking occasion for each of the alcoholic beverages of beer, wine, and spirits. The frequency questions were almost identical for all three alcoholic beverages, with “never” valued as 0, “every or every second day” as 250, “1–2 times a week” as 80, “a few times a month” as 12, and “more seldom” as 6. The questions for the amount of intake of alcohol on each occasion had 7–9 response alternatives for each beverage. The scores for these responses are presented in Additional file 1: Table S1. For each of the beverages, there were also weights corresponding to the alcohol percentage – 0.05 for beer, 0.14 for wine, and 0.40 for spirits. The total alcohol consumption was calculated as the sum of alcohol intake for each beverage. For each beverage, the alcohol intake during a year was calculated by multiplying the frequency score, the amount score, and the weight. The alcohol intake score has been used in previous studies of the Northern Swedish Cohort and is considered to work well [7]. An alcohol intake score below 140 was used as the reference value for our analyses.

Cutoff-values for the AVSI, AVAT, and alcohol intake were chosen to build two groups containing approximately equal numbers of individuals in both. Body mass index (BMI) was derived from self-reported weight and height and calculated as weight/height^2^. Those with BMI ≥ 30 kg/cm^2^ were defined as obese and those with BMI between 25 and 30 kg/cm^2^ were defined as overweight, and the two groups were used as the exposed groups.

### Statistics

Logistic regression, G-computation, and a method using propensity scores were used to analyze the effect on health from unemployment. The logistic regression model studies the effect of unemployment on health by comparing groups, and it is the most commonly used method for studies of the relationship between unemployment and health [2]. The other two methods estimate the risk difference using counterfactual arguments. The risk difference is estimated with E[Y(1)] − E[Y(0)], where E[Y(1)] corresponds to the expected effect if all individuals are unemployed and E[Y(0)] corresponds to the expected effect if all individuals are employed. Thus, the advantage with these methods estimators are that they correspond to the marginal effect of becoming unemployed.

The procedure for our analysis was to first include all identified potentially confounding variables in a *full* model. Thereafter we applied a *reduced* model using only the significant variables in the full model. In the reduced model, we used the same participants as in the full model to allow for comparisons between models, which meant that 15 individuals with complete information for variables in the reduced model were excluded in these analyses. Interactions between variables were not considered in our analyses. We tested models that included and excluded our candidate variables in the reduced and full model, but these only had a limited effect on the estimate of unemployment on health. We therefore included education level, marital status, and occupation in the reduced and full models despite these potentially being collinear variables.

Propensity scores were introduced in 1983 by Rosenbaum and Rubin [24], but these have rarely been used to study the effect of unemployment on health [2]. The propensity score is the conditional probability of being assigned to the exposure group based on baseline covariates. This implies that an exposed and unexposed individual with the same propensity score should have had the same probability of being exposed. If the estimates of the propensity score are unbiased, which cannot be assumed, these groups would then correspond to those in a randomized controlled trial (RCT). The bias of the estimates depends on how well the propensity score can balance both measured and unmeasured confounders. The strength of the RCT is that the balance of confounders can well be accomplished due to the randomization if the study protocol is followed, something that an observational study cannot accomplish in the study design.

The inverse probability weight (IPW) was defined as $w=\frac{X}{PS}+\frac{1- X}{1- PS}$, where *X* refers to the exposure (employed/unemployed), and *PS* to the estimate of the propensity score. We used an IPW estimator, as suggested by Lunceford and Davidian [25], to estimate the risk difference

$$ {RD}_{IPW}=\frac{1}{n}\left[\sum_{i=1}^n{Y}_i\left(\frac{X_i}{PS_i}-\frac{\left(1-{X}_i\right)}{1-{PS}_i}\right)\right], $$

where *Y* refers to the outcome (self-rated health). The marginal effect from this estimator corresponds to the average treatment effect [26]. In our study, logistic regression, with covariates from the statistical model, was used to estimate the propensity scores, which correspond to the probability of being unemployed for an individual based on his or her characteristics.

We calculated the standardized difference for each covariate in the reduced model to assess the balance of covariates between the employed and unemployed group, both with and without a weight [27]. For the unweighted sample the standardized difference was defined as

$$ d=100\frac{\left({\widehat{p}}_{unemployed}-{\widehat{p}}_{employed}\right)}{\sqrt{\frac{{\widehat{p}}_{unemployed}\left(1-{\widehat{p}}_{unemployed}\right)+{\widehat{p}}_{employed}\left(1-{\widehat{p}}_{employed}\right)}{2}}}, $$

where the denominator is the pooled standard deviation and $\widehat{p}$ is the estimated proportion of exposed individuals for the covariate. For categorical variables with three outcomes, two dummy variables were created and the reference group for the covariate was set to 0. For the weighted sample, the pooled standard deviation was calculated with

$$ \sqrt{\frac{\sum {w}_i}{{\left(\sum {w}_i\right)}^2-\sum {w}_i^2}\sum {w}_i{\left({x}_i-{\overset{-}{x}}_{w eight}\right)}^2,} $$

where ${\overset{-}{x}}_{w eight}=\frac{\sum {w}_i{x}_i}{\sum {w}_i}$ is the weighted prevalence for the covariate, and $\widehat{p}$ was calculated with ${\overset{-}{x}}_{w eight}=\frac{\sum {w}_i{x}_i}{\sum {w}_i}$, but for employed and unemployed groups separately.

G-computation is a similar regression method as logistic regression, but it differs in that it aims to estimate marginal effects [28]. For the G-computation, logistic regression was first performed with all variables in the statistical model, including labor market status. The risk difference was thereafter estimated based on the logistic regression as the difference between the expected effect if all individuals are unemployed and the expected effect if all individuals are employed. Results from our G-computation estimator can be directly compared with those from the IPW estimator.

We performed stratified analyses for the variables in the reduced model. We also performed stratified analyses for men and women because it has been shown in several studies that the effect of unemployment on health differs for the two groups [2]. In some cases our stratified analyses used smaller samples than has been recommended for logistic regression [29], which is at least 10 events per variable for the less-common outcomes. Such situations only occurred rarely for the logistic regression when self-rated health in 2007 was used as the outcome variable, but it became more of a problem when labor market status was used as the outcome variable for the estimation of propensity scores. We have highlighted in the results section when this criterion was not fulfilled.

R Studio was used for all statistical analyses (R Studio, Boston, MA). The GLM procedure in R was used for logistic regression estimates, and confidence intervals for the estimator were derived with the profile likelihood [30]. The Bootstrap technique with replacement was used to derive estimates of the mean square error for the IPW and G-computation estimators [31]. The 2.5% and 97.5% percentiles of the Bootstrap simulations were used to calculate the 95% confidence intervals. Sensitivity analyses were performed for the exclusion criteria of no unemployment during follow-up. In the first analysis, no individuals were excluded due to unemployment during the follow-up. In the second analysis, a variable was introduced with those defined as having unemployment during the follow-up as the exposed group and those without unemployment as the reference group. Pearson’s χ^2^-test was used to test if the exposure variable (labor market status) was associated with potential confounders. Statistical significance was defined at the 5% level.

## Results

### General characteristics

Of the 620 individuals without self-reported unemployment from 1995 to 2007 who had information for all study variables, 98 (16%) were defined as unemployed in 1995. The characteristics of the study population are presented in Table 1. It is notable that most participants reported good self-rated health in 1995 among both the employed (81%) and unemployed (74%), while the self-reported health for both groups had worsened by 2007, with this being more pronounced among the unemployed. Labor market status had an association with self-rated health in 2007, cash margin, and smoking (Table 1).

**Table 1:** Characteristics for the study population (*n* = 620)

	Employed (*n* = 522)		Unemployed (*n* = 98)	
Self-rated health 2007*	*n*	%	n	%
*Poor*	159	30%	43	44%
*Good*	363	70%	55	56%
Self-rated health 1995				
*Poor*	100	19%	25	26%
*Good*	422	81%	73	74%
Education level 1995^a^				
*Secondary education*	209	40%	46	47%
*Upper secondary education*	90	17%	22	22%
*University*	223	43%	30	31%
Marital status 1995				
*Married*	396	76%	72	73%
*Single*	126	24%	26	27%
Occupation 1995				
*Blue-collar*	202	39%	44	45%
*Low white-collar*	85	16%	18	18%
*Medium–high white-collar*	235	45%	36	37%
Gender				
*Man*	289	55%	49	50%
*Woman*	233	45%	49	50%
Availability of Social Integration (AVSI) 1995				
*Low*	166	32%	37	38%
*High*	356	68%	61	62%
Availability of Attachment (AVAT) 1995				
*Low*	248	48%	51	52%
*High*	274	52%	47	48%
Cash margin 1995*				
*Access*	441	84%	73	74%
*No access*	81	16%	25	26%
Smoking 1995*				
*Not smoking*	388	74%	61	62%
*Smoking ≤ 10 cigarettes*	88	17%	25	26%
*Smoking > 10 cigarettes*	46	9%	12	12%
Alcohol intake 1995				
*Low*	270	52%	43	44%
*High*	252	48%	55	56%
Body mass index 1995				
*Normal*	339	65%	54	55%
*Overweight*	154	30%	37	38%
*Obese*	29	6%	7	7%

* Significance at 5% level using χ^2^-test

^a^ Secondary education corresponds to at most 2-years of secondary education, and upper-secondary education corresponds to 3–4 years of secondary education

Note: Self-reported health is presented for both 1995 and 2007, and other variables are presented only for 1995

### Long-term effect on health from unemployment

Our results showed a clear negative health effect from unemployment regardless of which estimator and statistical model we used (Table 2). The crude odds ratios and the adjusted odds ratios for the full and reduced models, and thereby the contribution from potential confounding variables, are presented in Additional file 2. The coefficients for the variables in the logistic regression were similar in both models, with the odds ratio for labor market status differing by only 0.01 units between the models. The differences between estimates for G-computation and IPW were also small between models. The standardized difference ranged from 5.5% to 17% without IPWs and from 0.71% to 2.1% when IPWs were used (Additional file 3: Table S3). Thus, the balance in observed baseline covariates was good for the propensity scores (Austin and Stuart referred to a standardized difference below 10% as being a level some authors considered to be negligible imbalance [27]).

**Table 2:** Long-term effect of unemployment at 28–30 years of age on self-rated health (*n* = 620)

		Model		
Method	Estimate	Full	Reduced	Crude
Logistic regression	Odds ratio	1.73	1.74	1.78
	Confidence interval	1.07–2.8	1.08–2.6	1.15–2.8
G-computation	Risk difference	0.111	0.113	-
	Mean square error	0.0029	0.0027	-
	Confidence interval	0.025–0.199	0.029–0.200	-
Propensity scores, inverse probability weighting	Risk difference	0.114	0.103	-
	Mean square error	0.0038	0.0012	-
	Confidence interval	0.018–0.220	0.016–0.188	-

The *p*-value is less than 0.05 for all effect estimates in the table. Analyses in the reduced models controlled for education level, marital status, self-rated health 1995, and occupation. The full model also controlled for gender, social network, cash margin, smoking, alcohol intake, and body mass index. Crude refers to the estimate with unemployment as the only predictor. Estimates represent the health effect on unemployed compared to employed individuals.

All results from our sensitivity analyses also showed a clear negative and significant health effect from unemployment. The sensitivity analysis without exclusion of the unemployed during the follow-up period gave an odds ratio with logistic regression of 1.85 and an absolute difference estimate of 0.129 with G-computation and 0.123 with IPW. The sensitivity analysis where individuals was grouped based on having had an employment spell or not during the follow-up period (instead of excluding individuals with unemployment spells) resulted in an odds ratio with logistic regression of 1.70 and an absolute difference estimate of 0.112 with G-computation and 0.106 with IPW, which were close to the estimates for the reduced model (odds ratio of 1.74 and absolute difference estimates of 0.113 (G-computation) and 0.103 (IPW)).

### Long-term effect of unemployment on health in groups of individuals

All of our stratifications showed a negative health effect for the unemployed compared to the employed, but not all of these were significant (Table 3). For the stratifications of education level, it was only for those with upper secondary education that there was a significantly poorer long-term health outcome for the unemployed compared with the employed for all estimates, while for both secondary education and university studies, there were significant negative effects only for the G-computation. For singles, there was significantly poorer health for the unemployed compared to the employed for the counterfactual estimates but not for the logistic regression estimate. For those who were married, the reductions in health among the unemployed were non-significant for all estimators.

**Table 3:** Long-term effect of unemployment at 28–30 years of age on self-rated health at age 42 for groups of individuals (*n* = 620)

	Logistic Regression		G-computation	Propensity scores, inverse probability weighting
	Odds ratio	Confidence interval	Risk difference	Risk difference
Education level^a^				
*Secondary education (n = 255)*	1.45	0.60–3.32	0.074*	0.052
*Upper secondary education (n = 112)*	5.99	2.03–19.3^b^	0.345*^b^	0.372*^b^
*University (n = 253)*	1.22	0.59–2.49	0.039*	0.013^b^
Marital status				
*Married (n = 468)*	1.58	0.90–2.74	0.069	0.072
*Single (n = 152)*	2.46	0.98–6.34	0.194*	0.211*^b^
Self-rated health 1995				
*Poor (n = 125)*	1.75	0.65–5.11	0.109*	0.169^b^
*Good (n = 495)*	1.77	1.02–3.01	0.116*	0.089
Occupation				
*Blue-collar workers (n = 246)*	1.56	0.74–3.25	0.087*	0.099
*Low white-collar workers (n = 103)*	1.51	0.49–4.54^b^	0.083*^b^	0.058^b^
*Medium–high white-collar workers (n = 271)*	2.29	1.04–4.96	0.169*	0.119
Gender				
*Man (n = 338)*	1.35	0.68–2.64	0.060*	0.056
*Woman (n = 282)*	2.29	1.15–4.55	0.168*	0.155*

* *p*-value below 0.05

^a^ Secondary education corresponds to at most 2-years of secondary education, and upper-secondary education corresponds to 3–4 years of secondary education

^b^ Logistic regression was used with fewer than the recommended 10 outcomes per variable for the least-occurring outcomes

Analyses controlled for education level, marital status, previous health status (self-rated health in 1995), and occupation, excluding the factor for which the stratification was done. Estimates represent the effect on unemployed compared to employed individuals

For both stratifications on self-rated health in 1995, there was significantly poorer health for the unemployed compared to the employed for the G-computation estimate, while this was only the case for those with good self-rated health in 1995 for the logistic regression estimator and for none of the groups with the IPW estimator. However, the logistic regression estimators were very similar numerically, and the difference in sample size is likely to explain why only one of them was significant. For occupation, it was only for medium- and high-level white-collar workers that statistically significant poorer health was observed for the logistic regression estimator, while statistically significant poorer health was seen for all groups for the G-computation estimator and for no groups for the IPW estimator. For the G-computation estimator there was significantly poorer health reported for both unemployed men and women, while the difference was only significant for women for the other two estimators.

## Discussion

In our study, we show in a 12-year follow-up of 30 year olds that there is a negative health effect from being unemployed at the age of 30 despite having had steady employment from the ages of 30 to 42 years. This provides evidence for a long-term effect from being unemployed at an older age than has previously been shown. Despite rather small samples (100–300 individuals), it is evident from our stratified results that the effect from unemployment differs between groups of individuals. Most pronounced is the long-term negative health effect for those with upper secondary education, those living alone, medium- and high-level white-collar workers, and women, while there was at least an indication of a negative long-term health effect of unemployment for all other groups.

Strandh et al. studied the long-term health effect from unemployment at the age of 30 from the same cohort for psychological symptoms, but could not confirm a long-term negative health effect [8], which is in contrast to our results. Their study used a different health measure, which probably explains the different results. Our findings of a larger negative health effect for women than men is in agreement with two previous studies from the Northern Swedish Cohort [19, 32], as well as with other Swedish studies [21, 33]. Contrary to these studies that had a short-term perspective on the health consequences from unemployment, our study investigated the long-term effect. It is an interesting finding, therefore, that women seem to be more disfavored from being unemployed both in the long- and short-term than men. There are still not any commonly agreed upon reasons as to why Swedish women seem to suffer more from unemployment than men. In applying Connell’s theory of gender relations on the Swedish labor market [20], it turns out that it is strongly gender segregated with women working in less paid occupations within the public sector and in worse work-environment [34, 35]. In addition, women work in more unsecure labor market positions and much more often than men they have part-time work. These could potentially be part of the explanation for our findings, and because it is important to find explanations behind the differences between unemployed men and women we recommend that more research is conducted on this topic from a gender theoretical perspective.

Few studies have presented results for health effects of unemployment for those with different education levels, and those that have looked at this have shown inconclusive results [2]. In our study, we showed a large negative health effect due to unemployment for those with upper secondary education, while there was a lower effect on health for those with secondary education and university education. Despite estimates with a potentially large bias due to few unemployed with an upper secondary education, this group’s health status seems likely to be more affected from unemployment in our study population. Also for occupation, only a few studies have presented stratified results [2]. Our study shows opposite results compared to these studies, indicating more health problems for the unemployed from a high-level occupation class than others. Stratified results for marital status have only been presented in two studies, and they showed no apparent difference between married and single individuals [12, 21]. Our study is, therefore, the first to show results indicating that single individuals might be more affected by unemployment than married individuals. Lack of a strong social network has been shown to be related to poorer health in two previous studies [16, 17], and perhaps our results could be interpreted as there being weaker social structure among single persons and that this has a negative effect on their health in relation to married people if they become unemployed. Differences in results for occupation and marital status between our study and others might be explained by us having a focus on the long-term effect from unemployment, which was not the case for previous studies. Although our study provides new and valuable information, more research is needed to be better informed about how education, occupation, and marital status are related to poor long-term health due to unemployment.

A strength with our study is the very low attrition rate (6%). Still, despite a large sample of 1010 participants at the latest follow-up, not all stratified results fulfilled the recommendation for logistic regression of at least 10 events per variable for the less-common outcome [29]. Thus, the stratified results might be unreliable and give biased results, both for these and other estimates where the sample size was only slightly above the recommended threshold. Nevertheless, our results on the group level are valuable from a descriptive point of view, even if they cannot give very strong statistical evidence. We restricted our selection of individuals to those without unemployment during the follow-up because we did not want a prolonged unemployment experience to affect our effect estimates. In our sensitivity analyses, results were similar when a variable for unemployment during follow-up was used, while not excluding individuals with unemployment during the follow-up gave a slightly stronger negative health effect. Thus, this selection criterion is likely to have little bearing on the estimates.

A negative long-term health effect due to unemployment might be related to precarious employment during the follow-up period. Of the unemployed with no unemployment during the follow-up in our study, 42 experienced precarious employment during the follow-up with only 15 of these experiencing it during the majority of the follow-up period. To avoid effect estimates that are mainly reflecting effects due to precarious employment, a further limitation of our study sample to only those without precarious employment during the follow-up period was an alternative. However, analysis based on such restriction would also require that precarious employment during 1992–1995 was used to define the employed group and a larger focus on precarious employment which was not the scope of our study. It would also result in a too small sample to have reliable estimates to restrict ourselves in such a way. We therefore considered our definition of the study sample and the labor market status groups to be the most feasible. If there is a non-negligible bias due to precarious employment during the follow-up period for our effect estimates then our conclusions are still likely to be valid, although they would then be indicative of unemployment being related to poor health due to future unstable labor market positions rather than due to the unemployment itself. It has been shown in studies from our study cohort that those with precarious employment has a poorer health than those with a stable employment [36, 37]. However, these studies have not evaluated the long-term consequences from having had a precarious employment, which is an angle that would be valuable to study and could be recommended for future research.

The idea with propensity scores is to create two groups in the same manner as an RCT and thus to avoid problems from confounding. For the overall long-term health effect due to unemployment, our results were similar to those from the G-computation that were derived using logistic regression. Similar results are an indication that both methods work well, and are also in line with previous comparisons between propensity score techniques and logistic regression [38], but unmeasured confounders can still cause problems for the estimates. Also for our third method, logistic regression, the conclusions were generally similar. The confidence intervals estimated from the non-parametric Bootstrap, which was used for the G-computation and the IPW estimators, were derived from small samples. The G-computation estimator had small confidence intervals for stratified estimates and might therefore give more significant results than there really are. On the population level, the sample sizes are large enough so the bias for the confidence intervals based on a non-parametric Bootstrap should be very small.

Health prior to unemployment might have been an important confounding factor to include in our analyses to limit the bias. We could have used information from earlier questionnaires (1981, 1983, and/or 1986) in the Northern Swedish Cohort. However, these questionnaires did not include self-rated health and were not informative about health close to the unemployment period (1992–1994) of our study. We did use self-rated health at the time of recent/current unemployment in order to explain how health has changed between the two occasions (1995 and 2007). Interestingly, our stratifications on self-rated health in 1995 showed very similar results. This indicates that health status at the time of becoming unemployed at most might play a small role in the long-term health experience from unemployment. This also gives good support for our results being highly reliable and not confounded by previous health. Avoiding unemployment therefore seems valuable from a public health perspective not only for those that suffer from poorer health at the time of the unemployment, but also for those whose health is at most marginally affected at the time of the unemployment.

In a previous review, it was shown that only gender, age, and geographical location are commonly reported on the group level despite results on the group level showing that the context matters for the extent to which unemployment leads to poorer health [2]. Our study supports the recommendation from this earlier review that there should be greater focus on results on the group level. In the review, it was also reported that it is common to include many factors in the statistical model for the analysis of the health effect due to unemployment in order to control for confounding effects, as is also the case with other social epidemiological research questions. Still, it is apparent that only a few, if any, of these factors are commonly analyzed on the group level. We therefore support the recommendation to increase the focus on the group level for our and other research questions within the social epidemiological research field.

We limited ourselves to self-rated health as the health outcome. It would be valuable to investigate the long-term health effect of unemployment on other health outcomes, e.g. somatic symptoms, especially because the study by Strandh et al. showed different results than ours. In future studies with similar research questions as ours, we propose that the G-computation and IPW estimators should be used more frequently because they present the marginal effect and not the relative effect like logistic regression. An article with a more thorough evaluation of the sensitivity to the model set-up, the impact from the definition of labor market status, and the similarity of estimates from G-computation and propensity scores with IPW is planned from the same material as in this article. These issues are highly important for the interpretation of the causal effect from unemployment on health.

Because there is a limited number of studies presenting results for groups of individuals, more research is needed to better understand for whom and to what extent unemployment is related to poor health. Still, studies based on small samples such as ours can provide valuable evidence for policy makers, and such studies from a public health perspective can help guide decisions on which labor market measures to prioritize and to whom they should be directed. It might, for instance, be recommendable to focus more on understanding why women seem to suffer more from unemployment than men and to potentially prioritize efforts that can improve their chances of being re-employed and thereby improving public health. Our results are also important to provide support for future studies that can confirm the relationships observed in this study. The study context has been shown to be highly relevant for research within unemployment and health, but we still think that our study will provide highly relevant information both for other areas within Sweden and in other countries.

## Conclusions

In comparison to young adults with employment, those who are unemployed suffer from poorer health not only shortly after their unemployment, but also later in life. Our study therefore implies that it is important to lower the unemployment rate during young adulthood from not only an economical, but also from a long-term public health perspective.

The health effect due to unemployment varies between groups. For future research, it is important to put more emphasis on identifying groups of individuals for whom unemployment is most related to ill health so that efforts can be put towards the groups with the greatest need.

## Additional files

Additional file 1: The questions and possible responses for amount of alcohol intake on each drinking occasion, with each response alternative translated to a value. (DOCX 12 kb)

Additional file 2: Unstratified estimates of the odds ratio for variables in the logistic regression models. (DOCX 14 kb)

Additional file 3: Diagnostics of the inverse probably weights for the reduced model. (DOCX 15 kb)

## Abbreviations

AVAT: Availability of Attachment
AVSI: Availability of Social Integration
BMI: Body mass index
IPW: Inverse probability weight
RCT: Randomized controlled trial
