# Supplementary material for: Is unemployment in young adulthood related to self-rated health later in life? Results from the Northern Swedish cohort
Source: BMC Public Health. 2017 May 30;17:529. doi: 10.1186/s12889-017-4460-z (PMC5450391; doi:10.1186/s12889-017-4460-z)
Supplement: Supplementary file 1 — The questions and possible responses for amount of alcohol intake on each drinking occasion, with each response alternative translated to a value. (DOCX 12 kb) [file 12889_2017_4460_MOESM1_ESM.docx]

**Table 1.** The questions and possible responses for amount of alcohol intake on each drinking occasion, with each response alternative translated to a value.

| **How much strong beer do you *approximately* drink on each occasion** | | **How much wine do you *approximately* drink on each occasion** | | **How much strong spirit do you *approximately* drink on each occasion** | |
| --- | --- | --- | --- | --- | --- |
| *Response* | *Value* | *Response* | *Value* | *Response* | *Value* |
| I never drink | 0 | I never drink | 0 | I never drink | 0 |
| A bottle or less | 33 | 2-5 cl | 4 | 2 cl | 2 |
| A can | 50 | 10-20 cl | 15 | 4-6 cl | 5 |
| Two cans | 100 | Half a bottle | 38 | 8-12 cl | 10 |
| 3-4 cans | 175 | 60 cl | 60 | 18 cl | 18 |
| 5-10 cans | 400 | A bottle | 75 | 37 cl | 37 |
| More than 10 cans | 700 | More than a bottle | 100 | 60 cl | 60 |
|  |  |  |  | 75 cl | 75 |
|  |  |  |  | More than a bottle | 100 |
