# Supplementary material for: Is unemployment in young adulthood related to self-rated health later in life? Results from the Northern Swedish cohort
Source: BMC Public Health. 2017 May 30;17:529. doi: 10.1186/s12889-017-4460-z (PMC5450391; doi:10.1186/s12889-017-4460-z)
Supplement: Supplementary file 2 — Unstratified estimates of the odds ratio for variables in the logistic regression models. (DOCX 14 kb) [file 12889_2017_4460_MOESM2_ESM.docx]

|  | **Labor market status** | **Education level** | | **Marital status** | **SRH 1995** | **Occupation** | | **Sex** | **Social network** | | **Cash margin** | **Smoking** | | **Alcohol** | **Body mass index** | |
| --- | --- | --- | --- | --- | --- | --- | --- | --- | --- | --- | --- | --- | --- | --- | --- | --- |
| **Model** | Unemp-loyed | Med | High | Single | Poor | Med | High | Male | AVAT  high | AVSI low | No access | ≤10 | >10 | High | Over-weight | Obese |
| **All variables** | 1.73* | 0.60 | 0.50* | 1.77* | 4.54* | 0.80 | 0.49* | 1.10 | 1.05 | 1.14 | 0.92 | 1.50 | 0.85 | 0.79 | 1.11 | 1.80 |
| **Significant variables in full model** | 1.74* | 0.60 | 0.51* | 1.75* | 4.48* | 0.78 | 0.49* | - | - | - | - | - | - | - | - | - |
| **Crude estimates** | 1.78 (1.15-2.8) | 0.80 | 1.02 | 1.74* | 4.54* | 0.88 | 0.61* | 0.94 | 1.02 | 0.97 | 1.45 | 1.69* | 1.45 | 0.89 | 1.23 | 2.33* |

**Table 1.** Unstratified estimates of the odds ratio for variables in the logistic regression models (n = 620)

* - p-value below 0.05; crude estimates refer to results from simple logistic regression; SRH – self-rated health
