# Supplementary material for: Is unemployment in young adulthood related to self-rated health later in life? Results from the Northern Swedish cohort
Source: BMC Public Health. 2017 May 30;17:529. doi: 10.1186/s12889-017-4460-z (PMC5450391; doi:10.1186/s12889-017-4460-z)
Supplement: Supplementary file 3 — Diagnostics of the inverse probably weights for the reduced model. (DOCX 15 kb) [file 12889_2017_4460_MOESM3_ESM.docx]

**Table 1.** Diagnostics of the inverse probably weights for the reduced model.

|  | **Unweighted^a^** | | | **Weighted^b^** | | |
| --- | --- | --- | --- | --- | --- | --- |
|  | *Difference in unemployment rate^c^* | *Standard deviation^d^* | *Standardized difference^e^* | *Difference in unemployment rate^c^* | *Standard deviation^d^* | *Standardized difference^e^* |
| **Education level** |  |  |  |  |  |  |
| *Secondary education (n = 255)* | - | - | - | - | - | - |
| *Upper secondary education (n = 112)* | 0.052 | 0.398 | 13.1% | 0.0049 | 0.388 | 1.27% |
| *University (n = 253)* | 0.069 | 0.495 | 14.0% | -0.0102 | 0.492 | 2.08% |
| **Marital status** |  |  |  |  |  |  |
| *Married (n = 468)* | - | - | - | - | - | - |
| *Single (n = 152)* | 0.024 | 0.435 | 5.50% | 0.0049 | 0.432 | 1.12% |
| **Self-rated health 1995** |  |  |  |  |  |  |
| *Good (n = 495)* | - | - | - | - | - | - |
| *Poor (n = 125)* | 0.064 | 0.415 | 15.3% | -0.0022 | 0.401 | 0.71% |
| **Occupation** |  |  |  |  |  |  |
| Blue-collar workers *(n = 246)* | - | - | - | - | - | - |
| Low white-collar workers *(n = 103)* | 0.021 | 0.378 | 5.51% | -0.0072 | 0.369 | 1.94% |
| Medium–high white-collar workers *(n = 271)* | -0.083 | 0.490 | 16.9% | 0.0060 | 0.497 | 1.20% |

^a^ Proportions in unweighted samples are available in Table 1.
^b^ Estimates after inverse probability weights based on the propensity scores have been applied to balance the groups.
^c^ The estimated difference between exposed and reference group. Reference group is mentioned in first row of the variable.
^d^ The standard deviation pools the unemployed (“treatment”) and employed (“control”) samples.
^e^ The absolute value of the standardized difference is presented.
